# Evaluation of regional water resources carrying capacity in China based on variable weight model and grey-markov model: a case study of Anhui province

**DOI:** 10.1038/s41598-023-40487-w

**Published:** 2023-08-18

**Authors:** Lyu Yan, Dong Jiao, Zhan Yongshi

**Affiliations:** School of Humanities and Social Sciences, Anhui University of Science & Technology, Huainan, 232001 People’s Republic of China

**Keywords:** Environmental sciences, Environmental social sciences

## Abstract

Water security is not only an ecological environmental issue but also a bearing on national security and development. The study of water resources carrying capacity is the basis for future socioeconomic development and is the driving force for social progress. Therefore, it is important to investigate the influence factors of regional and national water resources carrying capacity (WRCC) and predict the future trend development. In view of the regional water resources data of the past 10 years in Anhui province, China, the *Driving force Pressure State Impact Response Management* (DPSIRM) model framework is constructed and the entropy weight method and variable weight theory can be used to make a comprehensive evaluation of the WRCC. Based on the comprehensive evaluation value, a modified Grey-Markov combination forecast can be introduced to predict the local WRCC in the coming years. The study on account of the Anhui Statistical Yearbook, the Water Resources Bulletin, and the water resources data of the Forestry Bureau for the past 10 years shows that the WRCC of Anhui Province is weak from 2010 to 2013 and gradually strengthens from 2014 to 2019; the WRCC of Anhui Province is mainly correlated with the impact subsystem, the management subsystem, and the state subsystem. The combined projections reflect that the future WRCC of Anhui Province is in good condition. It is recommended that the Anhui provincial government should strengthen water security and management, improve water resources utilization techniques, and construct complete and effective management tools and measures to fundamentally safeguard the province's water resources security and improve the WRCC.

## Introduction

Human flourishing relies on water resources. They provide a guarantee of survival and development. In addition to being a natural resource, it also is a strategic socioeconomic resource that plays a key role in the development of human society^1^. Therefore, water security is not only a national security issue but also an ecological and environmental issue. As the population has expanded, economic development has accelerated and living standards have risen globally, cities have begun to need more water than they can supply, and water scarcity has become a major concern and obstacle to sustainable development^2^. It is estimated that by the middle of the century, a quarter of the world's population will live in countries with severe water scarcity and be at risk of water stress. One of the challenges of ecological security has been how to achieve sustainable development between ecological health, economic progress, and social progress^3^.

In the past few years, China has become one of the countries with the most severe water scarcity, with two-thirds of its cities suffering from varying levels of water scarcity. Due to the continuous growth of population, water shortage, the progress of water-saving technology and the improvement of people's awareness of water-saving, the per capita water consumption in most parts of China shows a downward trend^4^. During the past 70 years, China's total water consumption has grown rapidly, generating a fivefold increase over the total amount of water used in the past, and the rapid growth of industry and agriculture has contributed to the deterioration and pollution of the water environment^5^. There is an increasing conflict between rapid economic development and water conservation^6^. In order to solve the problem of water shortage, China has taken the following measures: First, inter-basin water transfer, such as the South-North Water Transfer Project, to reallocate water resources. The second is to build reservoirs for water storage. The third is to set up a competent department and make laws and regulations. Fourth, is water conservation, not wasting a drop of water. Advocate the environmental protection concept of "the silver mountain of gold is the green mountain of water". Vigorously popularize water-saving domestic water appliances, strive to create water-saving cities, and implement sustainable development. As a result, it becomes necessary to study WRCC in China's provinces and cities in order to promote socioeconomic development and maintain environmental security. WRCC research covers theoretical to empirical evidence, from "water-ecology-socio-economic" multidisciplinary fundamental issues to sustainable development issues, from hydrological and water resources science to socio-economic science, planning science and other different levels and disciplines of research scope, to WRCC is a study to measure whether the regional water resources system can be a reasonable scale of sustainable socio-economic development under a certain level of economic, social and scientific development, with the premise of ecological and environmental health development and socio-economic sustainable development coordination. The introduction of WRCC provides guidance for the future development of China's social economy.

It was the research in the field of ecology^7^ that led to the concept of carrying capacity and it has since been adapted to various disciplines such as environmental carrying capacity^8^, land carrying capacity^9^, agricultural carrying capacity^10^ and urban carrying capacity^11^ as a consequence of the conflict between population size and resource allocation. Yifeng Wang et al. established a water resource carrying capacity evaluation model, and evaluated the carrying capacity of water resources in Shendong mining area^12^. Xian Wu et al. used the slime mold algorithm (SMA) to optimize the allocation of water resources in Wuzhi^13^.Yan Han et al. established a model for the risk assessment of the WRCC by combining the fragility of the water resources carrying system with the compound damage posed by climate change, urbanization, and industrialization^14^. It was the Xinjiang Soft Science Project Group (1989) that introduced WRCC indirectly in 1989, as the impact of human activities intensified and the significance of water resources became ever more apparent. Experts and scholars have studied WRCC since its introduction. There have been studies that attribute WRCC to sustainable socioeconomic development in a regional environment^15^, while others have attributed it to the maximum amount of water resources a region can provide for human consumption^16^. To date, there has been no comprehensive definition of the concept of WRCC. For the purposes of this study, WRCC has been defined as the maximum capacity of industrial, agricultural, population, and urban scale that water resources can support without adversely affecting local water environments^17^. There is a comprehensive discussion of the relationship between water, economy, and ecology. In the academic community, it is also the most widely used definition of WRCC. In recognition of the increased economic impact of water scarcity and security issues, most studies have combined WRCC with theories of sustainable development, and research on WRCC has moved from the study of individual natural resources to the study of the relationship between water, ecology, and the economy^18,19^.

Water is a large and complex resource, and the carrying capacity is an important indicator for local sustainable development, which impacts urban and rural economic development. When constructing the WRCC indicator system, there are two main approaches: one is to divide the whole system into interconnected and independent subsystems based on system theory. The second option is to select evaluation indicators that use a fixed model, taking into account the economic, social, ecological, and environmental factors that affect the water resources system, so that the indicators can reflect better the connections and differences between the elements in the system. Lu et al. Used AHP to study the water environment carrying capacity of Huai'an City from 2005 to 2014^20^, Zhang et al. Used a coupled model to study the water environment carrying capacity of Guizhou Province^21^, and Yang et al. Predicted the development trend of WRCC in Xi'an City through SD model^22^. Gao et al. Analyzed the dynamic changes and influencing factors of WRCC in Hefei city^23^. Others have used the ecological footprint method to quantitatively assess water security^24^. The principal component analysis method has lower accuracy than the original dataset in the index standardization approach, and some uncertainties remain. The multiple objective analysis methods is impacted by its scale limitations, and the selection of appropriate parameter values is inadequate and better suited to smaller research areas. The artificial neural network method is difficult to achieve in practice while quantifying the evaluation results. The ecological footprint method is unable to provide detailed and specific assessments. Water resource systems are complex and they are affected by both natural and human factors. Numerous studies have been conducted on WRCC evaluation models, including the DPSIR model^25^, CN-AM model^26^, projection pursuit model^27^, system dynamics method (SD)^22^, clustering methodology^28^, and others^29^. Furthermore, numerous research methods have been carried out in this field, including the traditional trend analysis method^30^, principal component analysis^31,32^, entropy weight method^33^, artificial neural network method^34^, fuzzy comprehensive evaluation method (FCE)^35^, analytical hierarchy process (AHP)^20,36^, ecological footprint method^37,38^, and multi-objective programming method^39^.

Only focusing on static changes in indicators will result in less accurate evaluation results for the WRCC. Studies on water resources can not only predict resource degradation accurately but also safeguard the water environment as well as promote sustainable socioeconomic development. Yet few studies have attempted to predict how regional water resources will change. Although some scholars have predicted the water environment carrying capacity of the study area using models^5,40^, they ignore the effect of uncertainty and stochasticity on the prediction process, reducing its accuracy and reliability.As a means of overcoming these shortcomings, this study developed a DPSIRM framework model by utilizing a combination of the entropy weight method and variable weight theory to evaluate WRCC in the study area. In 2012, Yang et al. Proposed the DPSIRM (*Driving force Pressure State Impact Response Management*) model, which is the first conceptual model of management about "people" in Ref.^41^. Modeling social and economic problems between resources, the environment, and the environment is an important function of the DPSIR model. The model was derived from the redesign of the PSR model by the United Nations^42^, which provides effective model support to domestic and international scholars. Management aspects have been added to the DPSIRM model based on the DPSIR model. China, with its large population and emphasis on sustainable development in the country's economic and social development process, focuses on the balanced development of ecology and economy, making DPSIRM a suitable evaluation system for China's national conditions. The DPSIRM model framework adds the "management" module, which changes the single directionality of the original model and strengthens the linkage between the indicators so that each sub-module of the system forms a causal feedback loop with each other and clearly captures the causal relationship between the social, economic and ecological environment systems, which can be used to build the evaluation system of water resources carrying capacity. N Chai et al. established a fuzzy analytical hierarchical process (FAHP)-gray cloud clustering method by constructing a DPSIRM model to comprehensively evaluate the water environmental carrying capacity of 11 provinces in China^43^. D SUN et al. evaluated the long-term security of water resources in Guizhou by constructing the DPSIRM model framework, using the gray correlation method and the material element analysis method^44^. But it is undeniable that the complexity of the evaluation of the environmental system relies only on the six sub-system modules is relatively simple, and the construction and improvement of the system needs to be further strengthened in the subsequent research.

Using the entropy weight method as a research method, weights can be objectively assigned to indicators, avoiding the uncertainty of subjective weighting. The variable weight theory is a study of the dynamics of indicators, and for the purpose of evaluating the value of WRCC, a reasonable and accurate weighting assignment for dynamic indicators is desirable. Lastly, this study combines GM (1, 1) with Markov chains to build a systematic, comprehensive, and dynamic WRCC forecasting model. Initial forecasts of WRCC can be made using GM (1, 1), and Markov models can address the randomness and volatility of those forecasts, making corrections to achieve long-term forecasting. Based on the historical water inflow observation data of typical coal mines, Li, B. et al. established a coal mine water inrush prediction model based on the unbiased gray and Markov theory, and predicted the water inflow of typical coal mines to verify the accuracy^45^.On the basis of the traditional gray theory prediction, Dang Luo et al. proposed a pseudo-grey metabolic grey Markov model to deal with the pre-diction issue in which the original sequences are oscillation sequences. The rationality and validity of the model are illustrated by taking the Zhengzhou City of Henan Province as examples^46^.

## Materials and methods

### Study area

Anhui Province, with a total area of 140,100 square kilometers and a combination of temperate and subtropical monsoonal climates, is an instrumental part of the Yangtze River Delta's economic development strategy. It is bordered by rivers and seas, has an 800-mile riverine urban agglomeration and the Anhui River Economic Zone, and straddles three major waterways: the Huai River, Yangtze River, and Xin’an River. Anhui is located in East China and economically belongs to the eastern economic zone of China. Geographic location 114°54′–119°37′ East longitude, 29°41′–34°38′ North latitude. Located in the middle and lower reaches of the Yangtze River and the Huai River, the Yangtze River Delta hinterland, in the middle and east, along the river and the sea, east of Jiangsu, west of Hubei, Henan, southeast of Zhejiang, south of Jiangxi, north of Shandong, 450 km wide from east to west, 570 km long from north to south.

**Figure 1:**
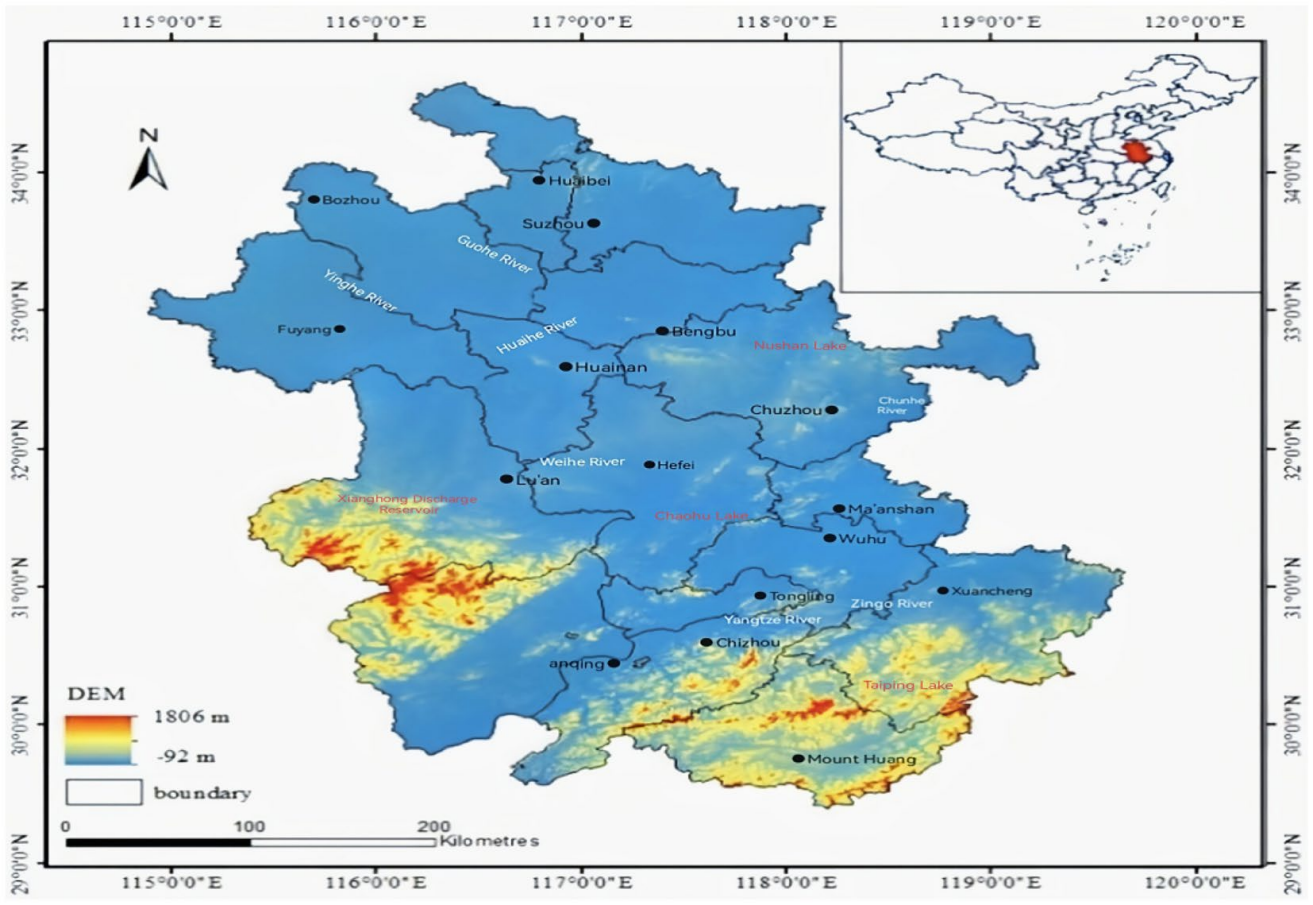
Map of Anhui Province.

There are more than 2000 rivers in Anhui Province, except for the southern Xin'an River system which belongs to the Qiantang River basin, the rest belong to the Yangtze River and Huai River basin. The Yangtze River enters the territory of Anhui Province from Hukou in Jiangxi Province to Wujiang in Hexian County and then flows into the territory of Jiangsu Province, running diagonally from southwest to northeast through the southern part of Anhui, 416 km in the province, belonging to the lower reaches of the Yangtze River, with a basin area of 66,000 square kilometers. The Yangtze River flows 400 km through Anhui, the Huai River flows 430 km through the province, and the Xin'an River flows 242 km through the province. There are more than 580 lakes in Anhui Province, with a total area of 1,750 square kilometers, 12 of which are large and 37 medium-sized. The lakes are mainly located along the Yangtze River and the Huai River, with an area of 1,250 square kilometers, accounting for 72.1% of the total area of lakes in the province. Among them, Chaohu Lake covers an area of 770 square kilometers and is the largest lake in Anhui Province and the fifth largest freshwater lake in China. Despite its proximity to river waters, Anhui Province is experiencing a severe water shortage, with only 848 cubic meters of water per capita in 2019. Water resources in the area are unevenly distributed, and ecological problems are relatively widespread, which poses a serious obstacle to Anhui's sustainable economic development. An overview of the study area is detailed in Fig. 1. The study area overview map of Anhui Province was drawn in ArcGIS 10.5 software, and the relevant data were obtained from the DEM elevation data in the Geospatial Data Cloud website(https://www.gscloud.cn/search).

### Data source

Data for the study were taken from the 2011–2020 “*Statistical Yearbook of Anhui Province*” “*Water Resources Bulletin of Anhui Province*”, and the “*Environment protection Bulletin of the Anhui Province*”. The authors were consulted for some missing data, and formulae were used to calculate the remaining data indicators that were not directly available.

G4 = total annual water consumption / 10,000 Yuan GDP.

G5 = Industrial water consumption / 10,000 yuan of industrial value added.

G6 = Total agricultural irrigation water use/total acres.

G12 = Area affected by regional drought + Area affected by floods.

G14 = Number of water functional areas meeting water quality standards/total number of water functional areas.

### Construction of index system

The DPSIRM model framework establishes the index system, uses the entropy weight method to determine the static weights, applies the variable weight theory to determine the dynamic weights, and calculates the comprehensive assessment value of WRCC to carry out the multi-dimensional evaluation of regional WRCC. Based on the multidimensional evaluation, a combined Grey-Markov prediction model should also be introduced to simulate and forecast the future carrying capacity of the region's water resources.

**Figure 2:**
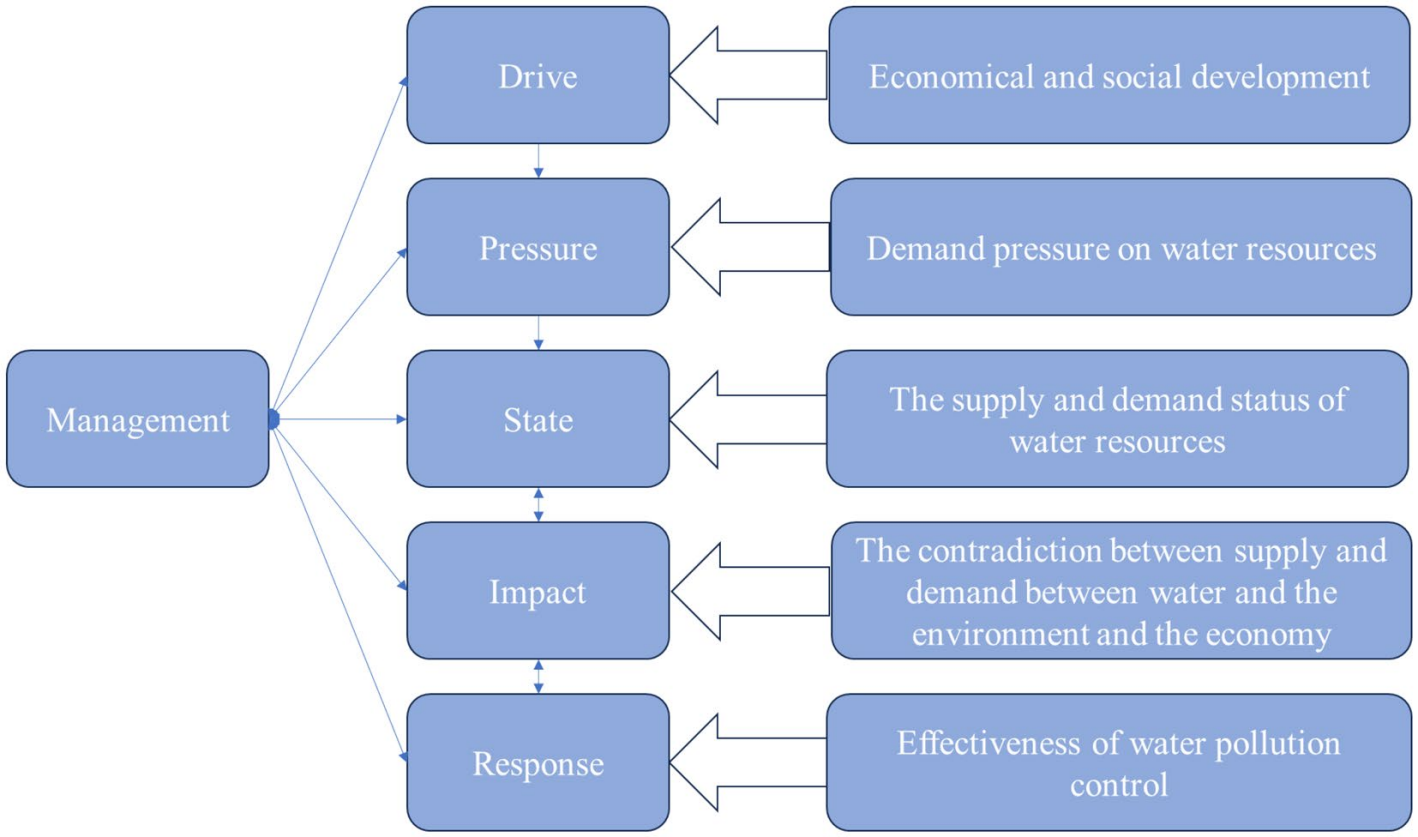
A causal network of DPSIRM model framework.

The DPSIRM model framework consists of six parts: driving force, pressure, state, impact, response, and management subsystems. As shown in Fig. 2. The construction of the WRCC indicator system based on the DPSIRM model framework and the significance of its selection is detailed in 0 (Tables 1, 2 and 3).

**Table 1:** WRCC indicators and their meanings.

Target layer	Indicator layer	Meaning	Attribute
Driving force (D)	Per capita GDP G1 (yuan/person)	Economic development status drives the carrying capacity of water resources	+
	Population density G2 (person/km^2^)	Population density as a driver of water resources	−
	Urbanization rate G3/%	Regional development drivers of water carrying capacity	−
Pressure (P)	Water consumption per 10,000 yuan GDP G4/m^3^	Measuring the pressure of economic development on the carrying capacity of water resources	−
	Water consumption per 10,000 yuan of industrial value-added G5/km^3^	Reflecting the pressure of industrial development on the carrying capacity of water resources	−
	Average acreage of water used for agricultural irrigation G6/m^3^	Reflecting the pressure of agricultural development on the carrying capacity of water resources	−
State (s)	Water resources per capita G7/m^3^	Per capita status of water resources	+
	Average annual precipitation G8/mm	A state that measures how much precipitation resources are available throughout the year	+
	Industrial ammonia nitrogen emissions per unit of GDP G9/(t/10^8^)	Measuring the state of industrial production on water resources	−
	Industrial COD emissions per unit of GDP G10/(t/10^8^)	Measuring the state of industrial production on water resources	−
Impact (I)	Forest coverage G11/%	The impact of surface water storage capacity on water resources	+
	Area affected by drought and floods G12/10^4^hm^2^	The impact of climatic factors on water resources	+
	Energy consumption per unit of GDP G13/(tce/10^4^ yuan)	Potential impacts of resource use on water resources	+
Response (R)	Proportion of water functional areas meeting water quality standards G14/%	Response reflecting water quality and safety	+
	Urban sewage treatment rate G15/%	Response reflecting water quality and safety	+
	Harmless disposal rate of domestic waste G16/%	Reflecting the response of domestic pollution treatment to the carrying capacity of water resources	+
Management (M)	Funding for wastewater treatment as a percentage of GDP G17/%	Reflecting the management of financial inputs for wastewater treatment	+
	Greenery coverage in built-up areas G18/%	Reflecting the management of town greenery construction	+
	Integrated utilization rate of industrial solid waste G19/%	Reflecting the management of industrial waste reuse	+

**Table 2:** Raw values of evaluation indicator data, 2010–2014.

Target layer	Indicator layer	2010	2011	2012	2013	2014
Driving force (D)	G1	21,923	27,314	30,683	34,256	37,184
	G2	487.29	490.79	492.65	494.58	495.07
	G3	22.71	22.93	22.89	22.92	22.69
Pressure (P)	G4	238.50	195.00	148.30	155.50	131.00
	G5	166.30	123.10	120.90	110.20	97.00
	G6	358.60	341.50	315.10	313.90	259.00
State (S)	G7	1578.20	1008.80	1170.60	971.20	1279.78
	G8	1308.9	1064.4	1173.8	1023.4	1278.5
	G9	3.3435	6.7424	5.5393	5.0185	2.3224
	G10	31.0269	58.5388	50.3934	43.8544	39.3257
Impact (I)	G11	27.5	27.5	27.5	27.5	28.7
	G12	207.1	22.79	179.89	209.26	71
	G13	0.71	0.63	0.60	0.57	0.53
Response (R)	G14	64.5	69.3	71.4	70.5	73.6
	G15	88.46	91.09	94.53	96.22	96.21
	G16	64.56	86.99	91.14	98.82	99.51
Management (M)	G17	0.14	0.11	0.11	0.11	0.10
	G18	37.5	39.5	38.8	39.9	41.2
	G19	84.6	78.7	81.5	84	84.4

**Table 3:** Raw values of evaluation indicator data, 2015–2019.

Target layer	Indicator layer	2015	2016	2017	2018	2019
Driving force (D)	G1	38,983	42,641	47,671	54,078	58,496
	G2	496.00	501.57	503.85	505.57	508.14
	G3	27.58	29.52	31.06	32.65	34.66
Pressure (P)	G4	131.20	120.50	105.50	95.20	74.80
	G5	96.80	94.00	80.10	78.10	74.30
	G6	282.40	284.70	275.30	265.90	249.70
State (S)	G7	1495.31	2018.23	1254.88	1321.68	848.07
	G8	1362.8	1612.7	1255	1314.7	935.8
	G9	4.0619	2.1401	1.9409	1.7436	5.2945
	G10	36.5530	18.8652	15.4871	15.4833	9.1297
Impact (I)	G11	28.7	28.7	28.7	28.7	28.7
	G12	94.89	125.99	39.81	86.4	96.46
	G13	0.52	0.48	0.44	0.39	0.37
Response (R)	G14	78.9	81.1	82.4	81.1	83.6
	G15	96.68	92.03	97.3	97.72	97.06
	G16	99.55	99.94	99.94	100	100
Management (M)	G17	0.11	0.09	0.09	0.08	0.08
	G18	41.2	41.7	42.2	42.5	42.72
	G19	88.5	82.6	90.9	88.1	78.6

Construction of DPSIRM Indicator System for WRCC. As shown in 0.

### Combined weighting method

#### Entropy weight method

The entropy weight method is an objective assignment method that uses the entropy of information to determine specific data, which is more accurate and closer to the real situation than subjective assignment methods.

Let the jth indicator of the ith sample of sample indicators be $x_{ij}$ (i = 1, 2, …, n; ‘n’ is the number of samples; j = 1, 2, …, m; ‘m’ is the number of indicators), First dimensionless the data, The formula is as follows:

For the positive indicators:

1 $$x_{ij}^{\prime} \, = \,\frac{{x_{ij} \, - \,m_{j} }}{{M_{j} \, - \,m_{j} }}$$

For the negative indicators:

2 $$x_{ij}^{\prime} \, = \,\frac{{x_{ij} \, - \,m_{j} }}{{M_{j} \, - \,m_{j} }},$$

$x_{ij}$’ is the standardized processing data, $M_{j}$ is the maximum of $x_{ij}$, $m_{j}$ is the minimum of $x_{ij}$.

The entropy method was used for the determination of the conventional weights of the indicators, with the following steps.

Calculating the characteristic weights:

3 $$p_{ij} = \frac{{x_{ij}^{\prime } }}{{\sum\limits_{i = 1}^{n} {x_{ij} } }}.$$

Calculating the entropy value:

4 $$e_{j} = - \frac{1}{\ln n}\sum\limits_{i = 1}^{n} {p_{ij} } \ln \left( {p_{ij} } \right),0 \le e_{j} \le 1.$$

Calculating the variability factor:

5 $$g_{j} = 1 - e_{j} .$$

Determining static weights $\omega_{j}$:

6 $$W_{j} = \frac{{g_{j} }}{{\sum\limits_{i = 1}^{m} {g_{j} } }},{\text{j}} = 1,2,3 \ldots \ldots {\text{m}}{.}$$

#### Variable weight theory

However, the entropy weight method is also deficient in that it determines the static weights of indicators, which does not accurately convey true information in the face of dynamic indicators. It is therefore necessary to introduce the variable weight theory to confirm dynamic weights to normalize the treatment of static and dynamic weights.

Wang (1985) pioneered the variable weight theory, and Some scholars proposed three forms of variable power: punitive, rewarding, and hybrid. Liu has refined the formulae of variation power theory^47^, resulting in a clear and unambiguous variation power formula. Li Considering that WRCC needs to be penalized for poorer indicators, it is necessary to identify the indicators that have a high degree of influence on WRCC and construct penalized state variable weight vectors^48^.

7 $$S_{j} (X) = \left\{ {\begin{array}{*{20}c} {e^{{ - g\left( {u_{i} - \gamma } \right)}} ,u_{i} \gamma } \\ {1,u_{i} > \gamma } \\ \end{array} } \right.,$$

*S*_*j*_*(X)* is the penalty variable weight.

In Eq. (7), *S*_*j*_*(X)* is the penalty variable weight “$g$”denotes the penalty factor and “$\gamma$”denotes the threshold for judging an alarm. Drawing on previous research results^49^, “$g$” takes the value of 0.81547 and “$\gamma$” takes the value of 0.85.

Calculating the penalty-type variable weights is as follows:

8 $$W(X) = \frac{{\omega_{j} S_{j} (X)}}{{\sum\limits_{j = 1}^{n} {\omega_{j} } S_{j} (X)}}.$$

In Eq. (8), $W(X)$ represents the variable weight of the indicator and $\omega_{j}$ represents the static weight of the indicator.

9 $$Z_{i} = \sum\limits_{j = 1}^{n} {\left( {W(X) \cdot {\mathbf{x}}_{ij}^{\prime } } \right)} .$$

In Eq. (9), *Z*_*i*_ is the comprehensive evaluation value of water resources carrying capacity.

### Grey-Markov prediction model

After determining the weights for the comprehensive evaluation of the regional WRCC, a prediction model is introduced to make grey forecasts of the future WRCC level. Considering that grey forecasts are prone to large errors for forecast values with time series, a Markov prediction model is added to correct for its volatility and combined to form a Grey-Markov prediction model. Firstly, a grey prediction model was used to make preliminary predictions of the carrying capacity of water resources and to determine the difference between the predicted and true values. Secondly, the Markov state transfer matrix is constructed; finally, the combined values predicted by the grey GM (1, 1) model are optimized by the Markov prediction model to produce the final combined predicted values.

The data were treated as grey quantities to obtain grey series, which were generated by a single accumulation using the GM (1, 1) model of the first-order differential equation, for the original series:

10 $$X^{(0)} = \left\{ {X^{(0)} (i),i = 1,2, \cdots ,n} \right\}$$

11 $$X^{(1)} (k) = \sum\limits_{i = 1}^{k} {X^{(0)} } (i) = X^{(1)} (k - 1) + X^{(0)} (k)$$

12 $$X^{(1)} = \left\{ {X^{(1)} (k),k = 1,2, \cdots ,n} \right\}.$$

The first-order differential equation for the GM(1, 1) model for the cumulative data.

13 $$\frac{{dX^{(1)} }}{dt} + aX^{(1)} = u,$$

$a$ is the developmental grey count and $u$ is the endogenous control grey count.

Propose $$\overset{\lower0.5em\hbox{$\smash{\scriptscriptstyle\frown}$}}{a} = \left( {\begin{array}{*{20}l} a \hfill \\ u \hfill \\ \end{array} } \right)$$, Using the least-squares method of solving.

$$\overset{\lower0.5em\hbox{$\smash{\scriptscriptstyle\frown}$}}{a} = \left( {\begin{array}{*{20}l} a \hfill \\ u \hfill \\ \end{array} } \right)$$

14 $$\overset{\lower0.5em\hbox{$\smash{\scriptscriptstyle\frown}$}}{a} = \left( {B^{T} B} \right)^{ - 1} B^{T} Y_{n}$$

15 $$B = \left[ {\begin{array}{*{20}c} { - \frac{1}{2}\left[ {X^{(1)} (1) + X^{(1)} (2)} \right]} & 1 \\ { - \frac{1}{2}\left[ {X^{(1)} (2) + X^{(1)} (3)} \right]} & 1 \\ \vdots & \vdots \\ { - \frac{1}{2}\left[ {X^{(1)} (n - 1) + X^{(1)} (n)} \right]} & 1 \\ \end{array} } \right]$$

16 $$Y_{n} = \left[ {\begin{array}{*{20}c} {X^{(0)} (1)} \\ {X^{(0)} (2)} \\ \vdots \\ {X^{(0)} (n)} \\ \end{array} } \right].$$

Then the time response function is

17 $$\hat{X}^{(1)} (k + 1) = (x^{(0)} (1) - \frac{u}{a})e^{ - ak} + \frac{u}{a}\,\,\,\,\,\left( {{\text{k}} = {1},{2}, \ldots ,{\text{n}}} \right),$$

18 $$\hat{Y}(k) = \hat{X}^{(0)} (k + 1) = \hat{X}^{(1)} (k + 1) - \hat{X}^{(1)} (k)\,\,\,\,\left( {{\text{k}} = {1},{2}, \ldots ,{\text{n}}} \right).$$

From the expression of the grey prediction model, it can be seen that the data curve of the model shows exponential changes, which will produce large data fluctuations and the data fitting effect is not ideal, so the Markov prediction model is introduced and amended to a Grey-Markov prediction model. Markov models are characterized by their discrete nature, where predictions of the future are only relevant to the present and not to the past and have a good degree of plausibility and accuracy. The process is as follows.

Status classification:

19 $$E_{i} = \left[ {\begin{array}{*{20}c} {a_{1i} ,a_{2i} } \\ \end{array} } \right]\,\,(i = 1,2, \cdots ,n)$$

$${\text{a}}_{1i} = \hat{Y}(t) + A_{i}$$

$${\text{a}}_{2i} = \hat{Y}(t) + B_{i}$$

$$A_{i}$$$$B_{i}$$ determined on a case-by-case basis.

Calculating the state transfer probabilities:

20 $$p_{ij} (k) = \frac{{n_{ij} (k)}}{{n_{i} (k)}}.$$

Calculating the state transfer matrix:

21 $$p(k) = \left[ {\begin{array}{*{20}c} {p_{11} (k)} & {p_{12} (k)} & \cdots & {p_{1n} (k)} \\ {p_{21} (k)} & {p_{22} (k)} & \cdots & {p_{2n} (k)} \\ \vdots & \vdots & \vdots & \vdots \\ {p_{n1} (k)} & {p_{n2} (k)} & \cdots & {p_{nn} (k)} \\ \end{array} } \right].$$

The predictive model was derived:

22 $$y(t) = \frac{1}{2}\left( {a_{1i} + a_{2i} } \right) = \hat{Y}(t) + \frac{1}{2}\left( {A_{i} + B_{i} } \right).$$

**Figure 3:**
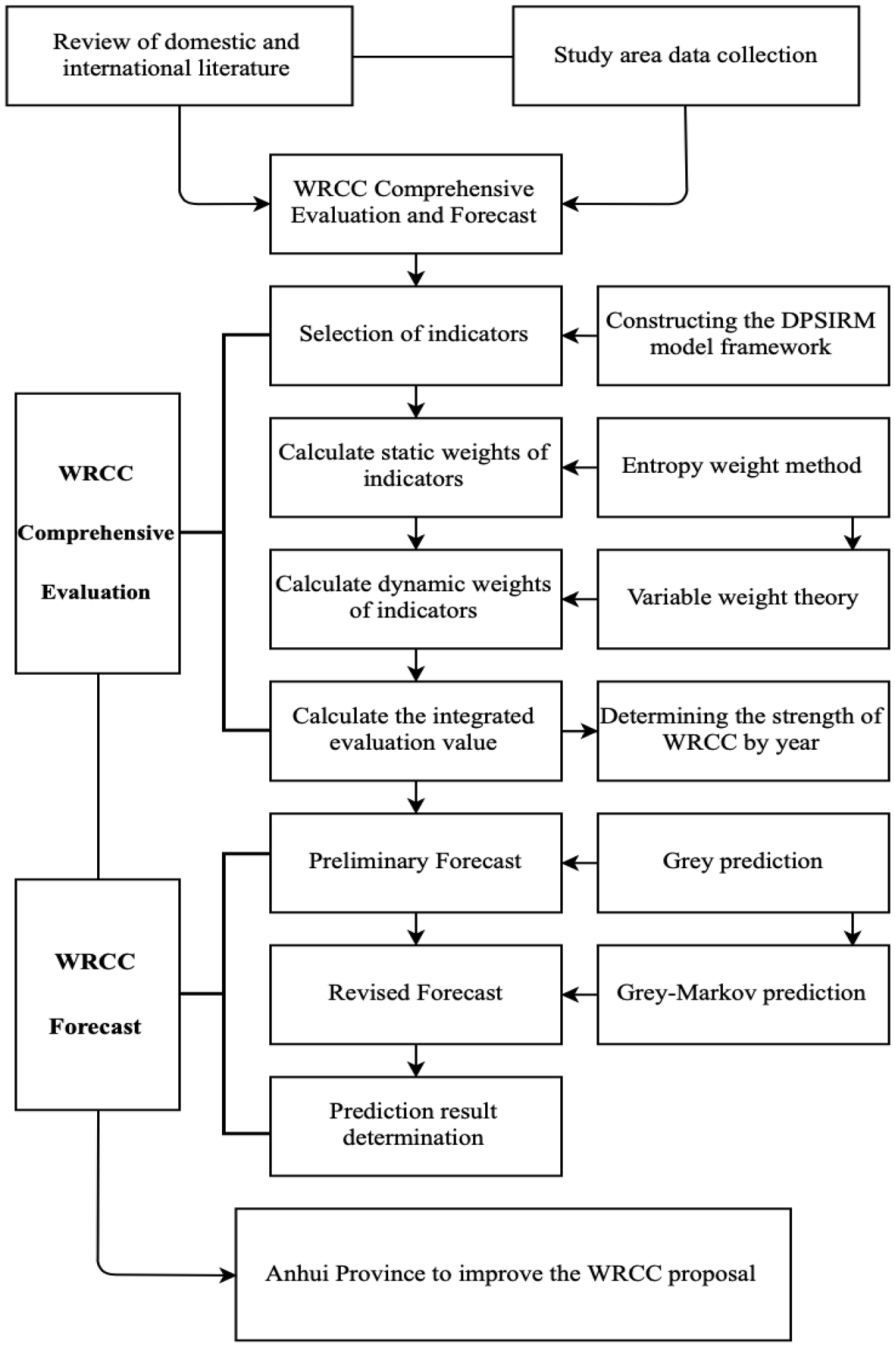
Method flow chart.

For a flow chart of the specific method see Fig. 3.

## Results

### Analysis of combination weighting results

#### Entropy weight method

The data from the processing of the indicator data using the extreme difference transformation method will have zero values. In consideration of the subsequent entropy calculation requirements and the elimination of the impact of zero values, this paper pans the data indicators to the right by 0.0001 units overall after the dimensionless transformation of the data. The data in Table 4 were calculated using Eqs. (1), (2), (3), (4), (5), (6) in the entropy weight method to obtain the following conventional weights for the indicators from 2010 to 2019.

**Table 4:** Conventional weights for each indicator.

Target layer	Indicator layer	Entropy value	Average constant weight
Driving force (D)	G1	0.8960	0.0534
	G2	0.8947	0.0541
	G3	0.9024	0.0501
Pressure (P)	G4	0.9323	0.0348
	G5	0.9407	0.0304
	G6	0.9140	0.0442
State (S)	G7	0.8763	0.0635
	G8	0.8989	0.0519
	G9	0.9078	0.0473
	G10	0.9035	0.0495
Impact (I)	G11	0.7784	0.1137
	G12	0.8710	0.0662
	G13	0.9157	0.0433
Response (R)	G14	0.9111	0.0456
	G15	0.9281	0.0369
	G16	0.9498	0.0258
Management (M)	G17	0.8428	0.0807
	G18	0.9221	0.0400
	G19	0.8665	0.0685

#### Variable weight theory

**Figure 4:**
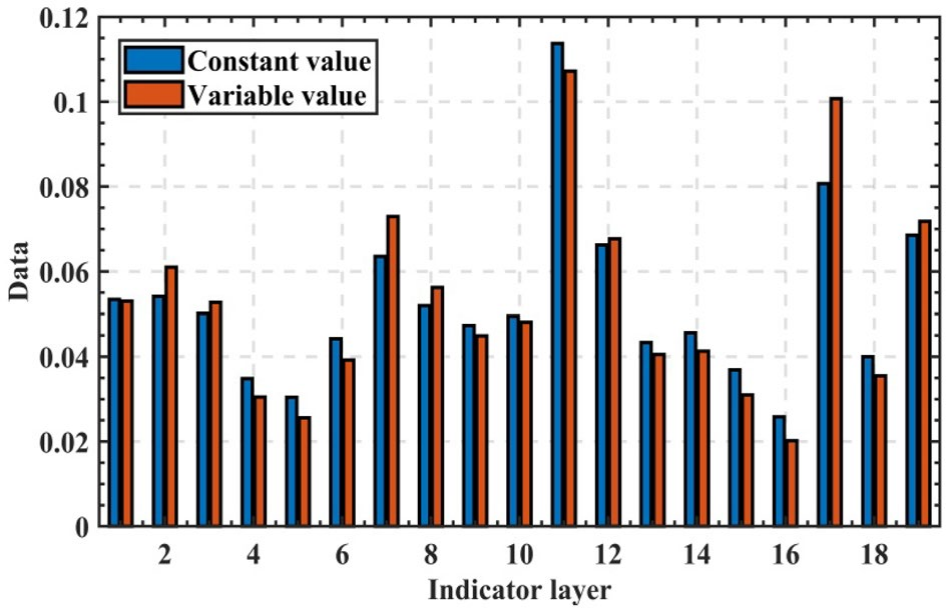
Graph of change in weights.

Dynamic weights were calculated for the indicator data using Eqs. (7),(8). See 0 and Fig. 4 for changes in the weights of the static and dynamic weights of the indicator data (Table 5).

**Table 5:** Changes in weights.

Target layer	Average constant weight	Average variable weight	Change rate/%	Indicator layer	Average constant weight	Average variable weight	Change rate/%
				G1	0.0534	0.0530	− 0.71
Driving force (D)	0.0525	0.0556	5.83	G2	0.0541	0.0610	12.87
				G3	0.0501	0.0527	5.20
				G4	0.0348	0.0305	− 12.31
Pressure(P)	0.0365	0.0318	− 12.85	G5	0.0304	0.0256	− 15.71
				G6	0.0442	0.0392	− 11.30
				G7	0.0635	0.0729	14.87
State(S)	0.0531	0.0555	4.66	G8	0.0519	0.0562	8.25
				G9	0.0473	0.0449	− 5.19
				G10	0.0495	0.0481	− 2.80
				G11	0.1137	0.1072	− 5.78
Impact(I)		0.0718	− 3.56	G12	0.0662	0.0677	2.16
				G13	0.0433	0.0405	− 6.48
				G14	0.0456	0.0413	− 9.43
Response(R)	0.0361	0.0308	− 14.58	G15	0.0369	0.0310	− 16.04
				G16	0.0258	0.0202	− 21.64
				G17	0.0807	0.1007	24.78
Management(M)	0.0631	0.0693	9.90	G18	0.0400	0.0355	− 11.26
				G19	0.0685	0.0718	4.73

In Table 4: After weighing the indicator data, the minimum variation of each indicator is 0.73% and the maximum variation is 29.63%. This proves that the dynamic weights obtained from the variable weight theory are an improvement over the entropy weight method and are more adaptable to the dynamic nature of the indicator data. As a result of the combined weighting calculation, the top five indicators are forest coverage (G11), funding for wastewater treatment as a percentage of GDP (G17), water resources per capita (G7), integrated utilization rate of industrial solid waste (G19) and area affected by drought and floods (G12). From the indicator layer weights, the average weights of forest coverage (G11) and funding for wastewater treatment as a percentage of GDP (G17) are larger, reflecting that the protection of natural resources and human intervention in environmental treatment are the key factors that become the influence of WRCC in Anhui Province. In terms of target-level weights, the impact subsystem has the highest average weight, followed by the management subsystem. In terms of the nature of the indicators, forest coverage (G11) has the highest average weight among the positive indicators, followed by the share of funding for wastewater treatment as a percentage of GDP (G17) and water resources per capita (G7) as the main factors for improving the WRCC. In addition to the high proportion of industrial water use in the total water demand, natural water shortages and rapid population growth also pose serious challenges. Among the negative indicators, the area affected by drought and floods (G12) has the highest average weight, followed by Population density (G2), both of which are the main threats to WRCC.

Using Eq. (9), we can arrive at a comprehensive assessment of the WRCC of Anhui Province for each year, as detailed in Table 5.

In Table 6, from the comprehensive evaluation of the WRCC of Anhui Province each year from 2010 to 2019 (Table 5), the overall trend of WRCC in these 10 years is increasing, indicating that under the current level of socioeconomic development, the WRCC of Anhui Province is increasing year by year, which can meet the sustainable socioeconomic development and ecological health development. The WRCC of Anhui Province in this decade can be roughly divided into two stages: the first stage (2010–2013) is relatively stable, with a weak WRCC. In the second phase (2014–2019) the WRCC level has increased significantly, and data shows significant changes in the indicators of the pressure subsystem in Table 6, the state subsystem, and the impact subsystem, with water consumption per 10,000 yuan GDP (G4), water consumption per 10,000 Yuan of industrial value-added (G5), industrial ammonia nitrogen emissions per unit of GDP (G9), industrial COD emissions per unit of GDP (G10) and area affected by droughts and floods (G12) decreased significantly. In the response and management subsystems, where there are human influences, the analysis of the indicator data shows a gradual increase in WRCC through positive human intervention. In 2019, Anhui Province's WRCC assessment value is slightly lower than that of previous years, and the data analysis shows that it is influenced by the state subsystem, with the water resources per capita (G7) and Average annual precipitation (G8) decreasing by 36% and 28.8% compared to the previous year; the area affected by drought and floods (G12) in the impact subsystem increases by 12% compared to the previous year, and the imbalance between supply and demand due to the increase in water demand in the ecological environment produces water resources in an In the management subsystem, the Integrated utilization rate of industrial solid waste (G19) was reduced, which reduced the WRCC.

**Table 6:** Combined assessment values by year.

Years	Overall assessment value
2010	0.4905
2011	0.2848
2012	0.4244
2013	0.4495
2014	0.6040
2015	0.6426
2016	0.6442
2017	0.6123
2018	0.6047
2019	0.4692

In conclusion, the WRCC of Anhui Province is mainly related to the impact subsystem, the management subsystem, and the state subsystem. On the whole, Anhui Province has achieved good results in reducing the contradiction between economic development and water shortage, achieving sustainable development in urban and rural areas, strengthening the rational use of water resources and waste management, etc. Relevant departments should do more to increase the number of water resources per capita, reduce water pollution, control water consumption, further optimize the allocation of water resources and promote water recycling, to strengthen the WRCC.

### Analysis of Grey-Markov prediction results

#### Calculating GM(1,1) model predictions values

The GM(1, 1) model is built with the comprehensive assessment value of WRCC of Anhui Province, and the cumulative series is generated as follows.

Original series:

$$X^{(0)}$$ = (0.4905, 0.2848, 0.4244, 0.4495, 0.6040, 0.6426, 0.6442, 0.6123, 0.6047, 0.4692).

Accumulate according to Eq. (11) to produce the series:

$$X^{(1)}$$ = (0.4905, 0.7753, 1.1997, 1.6492, 2.2532, 2.8958, 3.5400, 4.1523, 4.7570, 5.2262).

Constructed model matrix and calculated parameters.

According to Eqs. (15), (16), we get:

*B*=$$\left[\begin{array}{cc}-0.6329& 1\\ -0.9875& 1\\ \vdots & \vdots \\ -4.9916& 1\end{array}\right]$$ *Y* = $$\left[\begin{array}{c}0.2848\\ 0.4244\\ \vdots \\ 0.4692\end{array}\right]$$

According to Eqs. (13) and (14) we get:$a$ = -0.04479, $u$ = 0.406339, substituting the parameters, we can get the predicted value, see 0.

**Figure 5:**
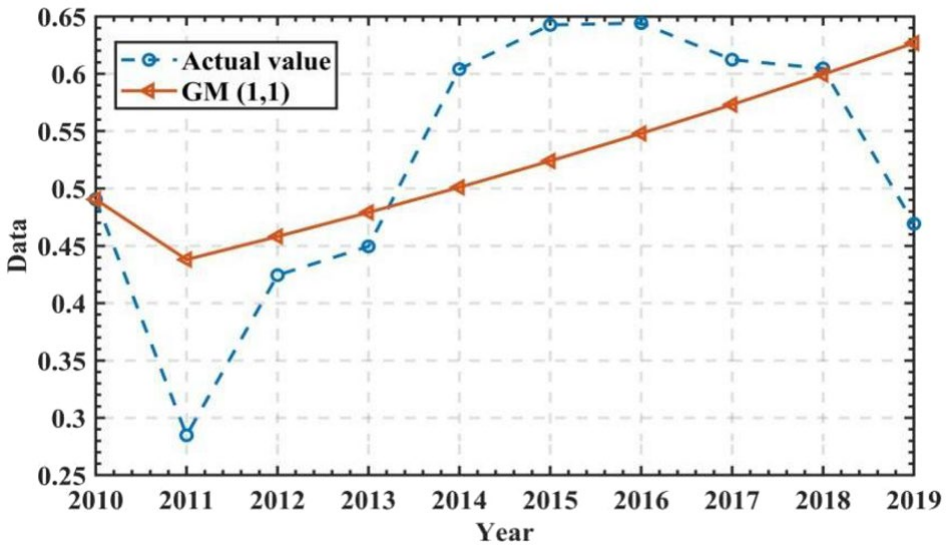
Comparison of actual values and GM (1,1) model predictions values.

Then, a graph comparing the trends in the combined assessment values and GM(1, 1) predictions is detailed in Fig. 5.

In Fig. 5: there is a discrepancy between the model predictions and the actual values of the comprehensive assessment of water resources in Anhui Province.

#### Calculating Grey-Markov predictions values

Based on the assessed values of WRCC of Anhui Province from 2010 to 2019, the relative values of the above comprehensive evaluation actual values and the predicted values of the GM(1,1) model were calculated, and the upper and lower limits of the relative values were used as the division interval to carry out the equal division of the interval distance, where the distribution area of the relative values was [0.65–1.23], and 0.65, 0.87, 0.99, 1.17 and 1.23 were selected respectively as the critical values. The above sample data were divided into four states to form Table 7. The state division of the forecast values from 2010 to 2019 is detailed in Table 8 (Tables 7, 8, 9, 10, 11, 12).

**Table 7:** Comprehensive assessment value of WRCC of Anhui Province calculated by GM(1, 1) model.

Years	Actual value	GM(1, 1) predicted value	Relative value
2010	0.4905	0.4905	1.0000
2011	0.2848	0.4380	0.6502
2012	0.4244	0.4581	0.9264
2013	0.4495	0.4791	0.9382
2014	0.6040	0.5010	1.2055
2015	0.6426	0.5240	1.2263
2016	0.6442	0.5480	1.1755
2017	0.6123	0.5731	1.0684
2018	0.6047	0.5994	1.0089
2019	0.4692	0.6268	0.7485

**Table 8:** Status classification table.

State number	Relative value	State
E1	0.65–0.87	0.65Y^(0)^ (1) – 0.87Y^(0)^ (1)
E2	0.87–0.99	0.87Y^(0)^ (1) – 0.99Y^(0)^ (1)
E3	0.99–1.17	0.99Y^(0)^ (1) – 1.17Y^(0)^ (1)
E4	1.17–1.23	1.17Y^(0)^ (1) – 1.23Y^(0)^ (1)

**Table 9:** Distribution of predicted value states.

Years	2010	2011	2012	2013	2014	2015	2016	2017	2018	2019
State	E3	E1	E2	E2	E4	E4	E4	E3	E3	E1

**Table 10:** State transfer matrix preparation forecast table.

Years	Initial state	Transfer step	State			
			1	2	3	4
2018	E3	1	2/3	0	1/3	0
2017	E3	2	2/9	2/3	1/9	0
2016	E4	3	2/9	2/9	7/27	8/27
2015	E4	4	14/81	1/3	5/27	25/81
Total			1.28	1.22	0.89	0.60

**Table 11:** Comparison of simulated predicted values for the two models.

Years	Actual value	GM(1, 1) predicted value	Residual	Grey-Markov prediction values	Residual
2010	0.4905	0.4905	0.0000	0.4905	0.0000
2011	0.2848	0.4380	− 0.1532	0.3329	− 0.0481
2012	0.4244	0.4581	− 0.0337	0.4260	− 0.0016
2013	0.4495	0.4791	− 0.0296	0.4456	0.0039
2014	0.6040	0.5010	0.1030	0.6013	0.0027
2015	0.6426	0.5240	0.1186	0.6288	0.0138
2016	0.6442	0.5480	0.0962	0.6576	− 0.0134
2017	0.6123	0.5731	0.0392	0.6190	− 0.0067
2018	0.6047	0.5994	0.0053	0.6473	− 0.0426
2019	0.4692	0.6268	− 0.1576	0.4764	− 0.0072

**Table 12:** Anhui Province WRCC 2020–2025 Integrated Assessment Value Forecast.

Years	2020	2021	2022	2023	2024	2025
Grey-Markov predicted value	0.6097	0.6376	0.8604	0.8998	0.9410	0.9841

Creating a state transfer matrix.

P(1)=$$\left[\begin{array}{cccc}0& 1& 0& 0\\ 0& \frac{1}{2}& 0& \frac{1}{2}\\ \frac{2}{3}& 0& \frac{1}{3}& 0\\ 0& 0& \frac{1}{3}& \frac{2}{3}\end{array}\right]$$ P(2) = $$\left[\begin{array}{cccc}0& \frac{1}{2}& 0& \frac{1}{2}\\ 0& \frac{1}{4}& \frac{1}{6}& \frac{7}{12}\\ \frac{2}{9}& \frac{2}{3}& \frac{1}{9}& 0\\ \frac{2}{9}& 0& \frac{1}{3}& \frac{4}{9}\end{array}\right]$$

P(3)=$$\left[\begin{array}{cccc}0& \frac{1}{4}& \frac{1}{6}& \frac{7}{12}\\ \frac{1}{9}& \frac{1}{8}& \frac{1}{4}& \frac{37}{72}\\ \frac{2}{27}& \frac{5}{9}& \frac{1}{27}& \frac{1}{3}\\ \frac{2}{9}& \frac{2}{9}& \frac{7}{27}& \frac{8}{27}\end{array}\right]$$ P(4) = $$\left[\begin{array}{cccc}\frac{1}{9}& \frac{1}{8}& \frac{1}{4}& \frac{37}{72}\\ \frac{1}{6}& \frac{25}{144}& \frac{55}{216}& \frac{175}{432}\\ \frac{2}{81}& \frac{19}{54}& \frac{10}{81}& \frac{1}{2}\\ \frac{14}{81}& \frac{1}{3}& \frac{5}{27}& \frac{25}{81}\end{array}\right]$$

Using the above state transfer matrix to prepare a forecast table.

A 4-step transfer probability matrix was used to calculate the state in which the WRCC assessment value of Anhui Province would be in 2019. Each element of the matrix is non-negative and the sum of the elements of each row is equal to 1. The elements are expressed in terms of probabilities and are mutually transferable under certain conditions. Some elements of a system are transferred in such a way that the nth result is only affected by the n-1th result, i.e., it is only related to the current state it is in and not to the past state.The four closest years to 2019 (2018, 2017, 2016, and 2015) were selected; four transfer steps of 1, 2, 3, and 4 were chosen to form a new probability transfer matrix, followed by probability summation in the newly derived matrix, with the largest result being the state the data in 0 should be in for 2019.

In 0, it can be seen that the 2019 data state E1 has the highest probability of being in the state where the integrated assessment value of WRCC of Anhui Province in 2019 is E1. Therefore, the Markov prediction value of the integrated assessment value of WRCC of Anhui Province in 2019 is:

y(t) = $$\frac{1}{2}$$(a_i1_ + a_i2_)*$${\hat{\text{x}}}$$(t)=$$\frac{1}{2}$$ (0.65 + 0.87) × 0.6268 = 0.4764.

The actual value of the 2019 comprehensive evaluation is 0.4692, the GM (1,1) forecast is 0.6268, with a relative error of 33.59%; the Grey-Markov forecast is 0.4764, with a relative error of 1.53%; the preliminary view is that the Grey-Markov forecast is closer to the actual situation than the grey forecast.

The predicted values derived from the GM(1,1) model was modified with the Markov model to obtain the Grey-Markov model predicted values for the comprehensive assessment of WRCC in Anhui Province, as shown in 0.

**Figure 6:**
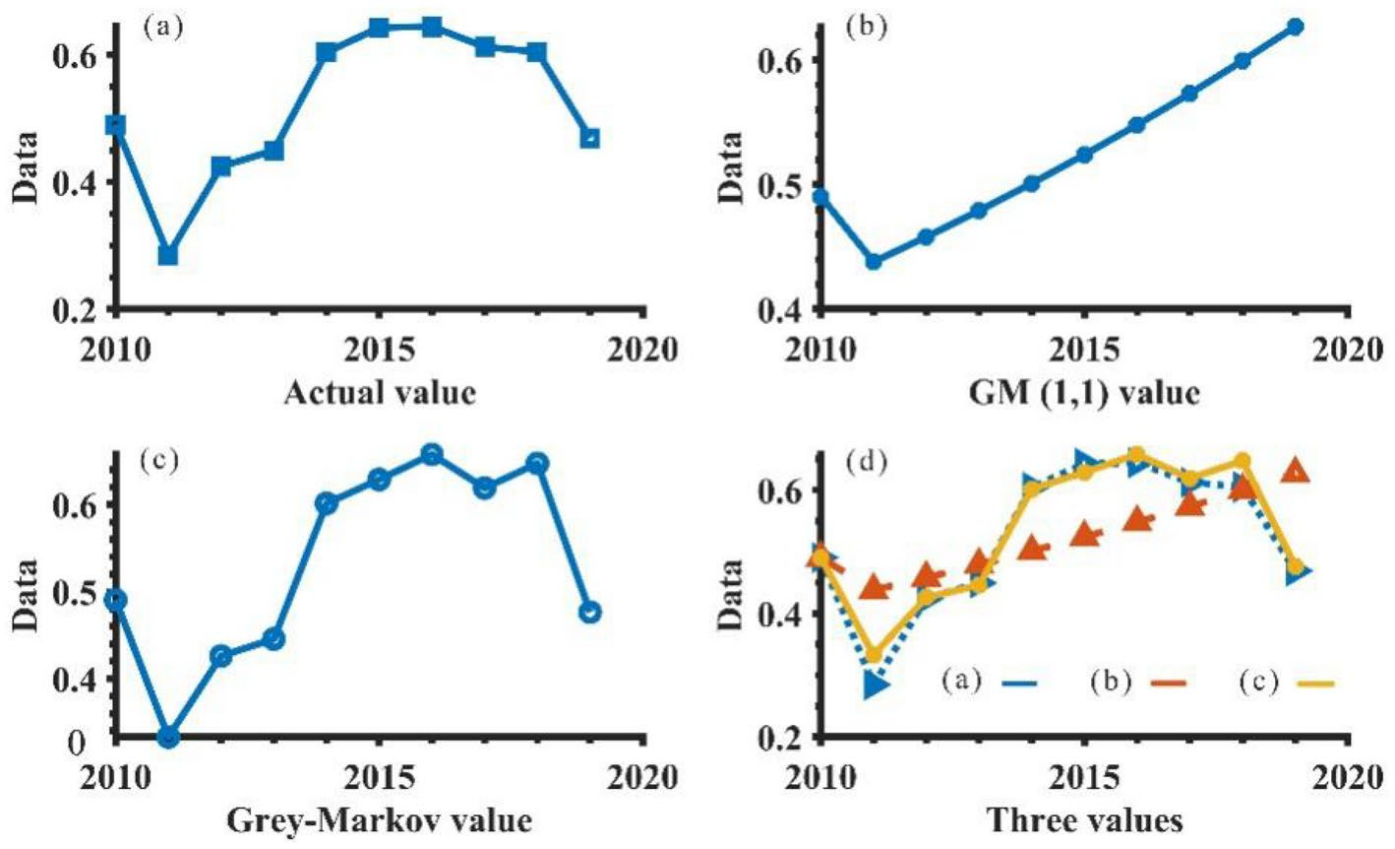
Comparison of the true value and the two predicted values.

In Fig. 6, comparison data trend graph, it can be seen that the predicted values simulated by the Grey-Markov model are closer to the actual values, with less relative error, and can be used to predict the comprehensive assessment value of WRCC in Anhui Province in 2020–2025 in 0.

## Discussions

In order to provide a theoretical basis for urban water resources management, the DPSIRM model was introduced to quantify the sustainability of water resources and identify key indicators that can be used as variables in water resources management measures or policies. The DPSIRM framework specifies six target layers and 19 data indicators related to the socio-economic factors affecting WRCC. By using Anhui Province as an example, the WRCC assessment methodology combines the entropy weight method, variable weight theory, grey prediction model, and Markov chain. This combination establishes a comprehensive WRCC evaluation and prediction model. Firstly, combining the entropy weight method and variable weight theory to determine weights minimizes the influence of subjective and objective factors. This compensates for the shortcomings of previous research based on a single weight and identifies the key factors which affect the development of WRCC. Secondly, Grey-Markov forecasts can be used to effectively predict stochasticity and uncertainty, which is very useful for long-term forecasting. Thirdly, the forecasting model is applicable even when WRCC fluctuatess temporarily. Additionally, assessments and predictions of WRCC impacts can be used to guide regional development and water conservation.

Driving force subsystem data indicates that the population density in Anhui Province in 2019 is 508 people per square kilometer and the urbanization rate is 40%, with rapid economic growth spurred by population growth. In the meantime, water demand and wastewater emissions are increasing, putting double pressure on the local environment. As the economy has grown and industrial facilities have improved, industrial indicators have appeared more frequently in the pressure, state, and impact subsystem. WRCC’s response and management subsystems indicate that financial and human investments in environmental management have greatly increased WRCC’s resilience and that the management subsystem should be given high priority for future development.

As a result of the combined weighting calculation, the top three indicators are forest coverage, funding for wastewater treatment as a percentage of GDP, and water resources per capita, The results show that the main source of impact on WRCC in Anhui Province is human activity, for which rational exploitation of resources and friendly protection of the environment should be increased. It has been shown that economic development can negatively impact water resources and the water environment, which is not conducive to the long-term sustainable development of water reserves. This is consistent with the concluding results of Ref.^50^.

Anhui Province did not pay enough attention to sustainable development before 2014, and economic growth dominated decision-making, which led to the overexploitation of water resources and degradation. The importance of WRCC has gradually been recognized, water allocation and use efficiency have been optimized, and water resources have been used more efficiently since 2014. Thanks to Anhui Province in 2014 completed the ten things of water conservancy, "Anhui Province to implement the Law of the People's Republic of China on Water and Soil Conservation (revised)" through the provincial people's Congress, the province's ability to govern water and manage water according to law continued to improve. Anhui Province implemented the "Water and Soil Conservation Law of the People's Republic of China" approach (revised) through the provincial people's Congress, and the province's ability to manage water in accordance with the law continued to improve. Issued "on deepening the implementation of water reform" and put into practice, the important areas of water and key aspects of the reform in an orderly manner. The provincial Development and Reform Commission, the provincial Department of Finance, and other 10 departments and bureaus issued the "implementation plan for the strictest water resources management system assessment in Anhui Province", the first time to carry out the strictest water resources management assessment work. The provincial government issued "Anhui Province small water conservancy project investment construction and management reform implementation measures", eight small water conservancy project renovation and upgrading achieved significant results. HuaiHe river water north transfer project started construction, key water conservancy project construction speed up and increase efficiency. Flood and drought relief benefits are significant, the annual flood and drought relief benefits amounted to 10.311 billion yuan, a strong guarantee of the safety of people's lives and property, to protect the stable and rapid economic and social development. Actively promote the "water ecological civilization city" and "water environment beautiful countryside" pilot work, water ecological civilization construction is effective. Strictly implement the main responsibility of the party committee and the discipline inspection committee to supervise the construction of the party wind and clean government, signed responsibility and commitment at all levels. Successfully held the fifth sports meeting of the provincial water conservancy department, the construction of spiritual civilization in depth. Management effectiveness significantly improved, and outstanding achievements. the emission of major pollutants has been effectively controlled, the environmental quality of key urban and rural areas has been improved, and the degradation of ecological functions has been curbed, supporting the province's good and rapid economic and social development at a minimal environmental cost.

Even with the fluctuations in WRCC from 2017 to 2019, the Grey-Markov prediction model has been able to forecast the trend of WRCC very accurately. There is a slow increase in WRCC in Anhui Province followed by a slow decrease, and then a continuous and steady increase. Grey-Markov predicted that no major changes will occur in the WRCC in Anhui Province over the next five years, which is consistent with this finding^40^. The future water resources score is generally within the safe range and has a tendency to steadily improve, but in order to have the carrying capacity to deal with emergencies, Anhui Province needs to increase the scientific protection of water resources, transform and upgrade the industry, and build a sustainable city.

The research object of WRCC is not a single factor, but a complex system with many complicated influencing factors. Although the WRCC indicator system constructed in this paper can better meet the needs of WRCC evaluation and prediction, there are still some shortcomings in the study, because there are many influencing factors of WRCC, the 19 indicators selected can only reflect WRCC changes to a certain extent, and cannot represent WRCC comprehensively, and there is still some room for improvement in terms of accuracy and precision. When constructing the indicator system, the entire process and influencing factors were simplified in order to highlight the main factors as well as to consider the availability of data, and the more important ones were selected for simulation analysis, and the completeness still needs to be improved. There is still some room for improvement in the research content and methods, and the weights obtained are still not precise enough, and more objective and cutting-edge model weight calculation methods can be used in the subsequent research. Finally, the prediction part of the paper is based on the smooth development of the current society, but there will be deviations in the actual life of the future society. In future research, a more scientific and objective method of prediction can be used to make the depth of the research go further and thus improve the practical application value.

## Conclusions

Based on the DPSIRM model framework, assessment and forecasting studies indicate that:

1. The impact subsystem of WRCC in Anhui Province is strongly influenced by three subsystems: the impact subsystem, the management subsystem and the state subsystem. The grey-Markov model is used to predict the future development trend of water resources recycling in Anhui Province. Although the water resources development situation has been improved in the future, the water resources security problem still needs to be paid attention to. The construction of the water resources management monitoring platform and the control of the red line of water resources development and utilisation should be strictly controlled.
2. Anhui Province should accelerate its transformation and upgrading to a service-oriented economy, and cities with a low level of manufacturing development should accelerate the transformation and upgrading of traditional manufacturing industries, strengthen technological innovation, renovate high water-consumption projects and improve the level of water safety and security. Cities should adjust the development focus of productive service industry according to regional industrial demand, and build a development pattern of benign interaction between manpower, manufacturing and productive service industry. Create water-saving cities, raise water-saving standards and enhance water-saving concepts. Increase investment in environmental management funds and manpower to encourage sustainable development of the local eco-economy and ensure the risk-resistant capacity of the WRCC in the future.
3. In the process of evaluating the Water Resource Recycling Centre (WRCC) in Anhui Province, it is only a comprehensive evaluation of the whole province, and the subsequent research can be specific to each city and targeted to strengthen it. In addition, the reliability of the evaluation model can be further verified by evaluating WRCCs in more different provinces and cities and different river basins. In the WRCC-driven correlation analysis, the influencing factors are obtained from the constructed WRCC evaluation index system, and it is necessary to obtain more sufficient information for more detailed analysis in the future research, to explore the WRCC mechanism in depth, and to dig out more influencing factors for correlation analysis.

## Data Availability

Some or all data, models, or codes that support the findings of this study are available from the corresponding author upon reasonable request.
